# Traumatic Metachronous Penile Fracture to the Contralateral Corpora

**DOI:** 10.1155/2022/6766741

**Published:** 2022-04-10

**Authors:** Christos Papaioannou, Savvas Omorphos

**Affiliations:** ^1^Barts and the London School of Medicine and Dentistry, Queen Mary University of London, London, UK; ^2^Department of Urology, Hippocrateon Private Hospital, Nicosia, Cyprus; ^3^University of Nicosia Medical School, Nicosia, Cyprus

## Abstract

Penile fracture is an uncommon condition in day-to-day urological practice. Though most cases of penile fracture are traumatic in nature, these are typically unilateral. Synchronous bilateral cases have been rarely reported. We present the third case recorded to date, to the best of our knowledge, of a metachronous penile fracture to the contralateral corpora due to trauma related to sexual intercourse. The first presentation demonstrated a significant tear to the left corporal body at surgical exploration that was repaired. There was no postoperative complications or erectile dysfunction on outpatient follow-up. Six months thereafter, the patient had another similar presentation and demonstrated a right corporal body fracture which was repaired surgically on an urgent basis. Prompt diagnosis and low threshold for surgical intervention are essential to reduce morbidity and prevent long-term complications.

## 1. Introduction

Penile fracture is defined as disruption of the tunica albuginea of one or both corpus cavernosum due to blunt trauma. It may be accompanied by partial or complete urethral rupture or injury to the dorsal nerves and blood vessels [1]. The classic symptom presentation is the triad of pain, rapid detumescence, and deformity, often with an audible “crack” or “pop” sound. Presentation may be delayed due to the psychological distress and embarrassment associated with such an injury, resulting in higher rates of long-term morbidity [2].

Tearing of the tunica albuginea is largely unilateral, though synchronous bilateral cases have also been reported [3]. Rarely, recurrent episodes of penile fracture have been documented though usually this is on the ipsilateral side due to scar rupture [4, 5]. More complex cases involve damage to the urethra as well [6, 7].

## 2. Case Report

A man in his 50s presented initially to the Accident and Emergency (A&E) department 15 hours following traumatic sexual intercourse in the missionary position, where he withdrew and hit his wife's bony prominence. He immediately heard a “pop” sound, followed by penile pain, swelling, discolouration, and loss of erection. He denied any urinary or bowel symptoms. Examination showed classical aubergine deformity confined to the whole length of the penis but normal scrotum and urethral meatus. Urinalysis revealed 1+ blood but was otherwise unremarkable.

He had a history of very mild asthma, with no regular medication or allergies. He had no urological history to note and no significant family history.

He was taken to the operating theatre on an urgent basis, which revealed a 5 cm tear in the left midshaft level of the tunica albuginea; urethra was intact. The haematoma was evacuated, and the tear was repaired using 8× interrupted sutures with 2-0 PDS. Artificial erection using saline solution revealed no leak or other microtears. Methylene blue confirmed this. Flexible cystoscopy showed the urethra was intact. In contrast to traditional teaching, no circumcision was performed at this time, due to the patient's preference. Postoperative period was uncomplicated, and he was discharged 72 hours following surgery, with the advice to avoid sexual intercourse for 8 weeks.

At outpatient review 3 months later, he reported normal, painless erections with no urinary symptoms, and he was discharged from follow-up.

He then presented 6 months later to the same A&E department 17 hours following a similar trauma during coitus. The patient described a “popping” sound, along with signs of swelling and rapid loss of erection; however, this time he denied any pain. Again, he reported no urinary or bowel symptoms. Examination revealed localized bruising to the right ventrolateral aspect of his penis. Tenderness was reported on palpation of the right base. Urinalysis was unremarkable.

Surgical exploration via circumferential incision revealed a 2 cm transverse tear to the right corpus cavernosum, with no damage to previous surgical wound or urethra. Following removal of haematoma, the defect was approximated with 2-0 PDS continuous sutures, and circumcision was performed. The dorsal neurovascular bundle was intact. Artificial erection was watertight, and flexible cystoscopy confirmed that the urethra was intact.

## 3. Discussion

During an erection, the penis is transformed from a safe, protected organ to one which is exposed and vulnerable to trauma. The tunica albuginea becomes significantly thinner, and intracorporal pressures increase dramatically which means that any added pressure from blunt trauma makes fracture more likely [8]. The most common point of fracture is along the ventral aspect of the corpus cavernosum as Buck's fascia splits in two [1].

Diagnosis is largely clinical, with most patients reporting the classical symptoms of pain, swelling, deformity, and rapid detumescence, often with a preceding “click” or “pop” [1]. Haematuria, blood at the meatus, and inability to void are suggestive of urethral injury. Pain is commonly not a presenting symptom; however, the painful sensation develops as haematoma develops and may indicate damage to the dorsal nerves. This highlights the importance of having a low threshold for considering diagnosis, particularly in the presence of relevant urological history. Examination may also vary but often shows the typical “aubergine sign,” in which there is swelling, ecchymosis, and penile deviation. In others, bruising and swelling may instead be confined to the scrotum.

In cases where the patient presents with haematuria or blood in the urethral meatus, an investigating tool should be utilised to assess the urethra of the patient. The latest European Association of Urology guidelines favour towards the use of flexible cystoscopy in these cases, due to the high false-negative rates associated with retrograde urethrography [9–11]. Furthermore, flexible cystoscopy could differentiate between partial or complete rupture of the urethra [12].

Most commonly, laceration is unilateral although synchronous bilateral cases have been documented in up to 20% of cases [3]. Urethral injury has been recognised in 10-20% of cases and is more likely if bilateral fracture has occurred [3]. Detailed review of literature revealed only 9 documented cases of recurrent penile fracture, with only two of these being contralateral [7, 13], as in this case. Nevertheless, periods between episodes of other patients in the literature were several years, as opposed to only 6 months with our patient.

There has been discussion regarding the use of absorbable vs. nonabsorbable sutures in repair of penile fractures. Use of nonabsorbable sutures has been advocated by some to reduce the risk of ipsilateral, same-site recurrent fractures though it is recognised that this can cause prolonged discomfort at suture-site, with knots palpable beneath the penile skin [4, 5]. It is believed that following surgical repair, fibrosis and scar tissue formation may result in an uneven distribution of tensile strength predisposing the contralateral side to injury, while rupture of these scars may cause ipsilateral fracture [4]. Interestingly, in our case, both repairs were performed using absorbable sutures (PDS), with a significantly shorter time period between presentation limiting collagen deposition and scar formation. Furthermore, due to the high incidence of postoperative penile curvatures, Dell'Atti et al. analysed the importance of intraoperative curvature correction in patients who presented with a cavernous body deviation. This could confer better quality of life and postoperative outcomes with the main disadvantage of a subjective loss of penile length [14].

Mechanism of injury also varies dependent on geographical location [1, 15]. In the UK and Western cultures, the most common cause of penile fracture is following sexual intercourse, where the erect penis accidentally hits their partner's perineum. One study showed that the greatest risk was posed while the woman was on top, while missionary position posed the smallest risk [2]. This is believed to be because men are able to respond to painful stimuli and prevent further damage from occurring. However, other mechanisms of injury are recognised, such as bending of the erect penis to void or to hide the erection due to sociocultural inhibition [7].

The current gold standard for treatment is recognised as early surgical intervention, compared to previous conservative measures such as compression bandages, cold compresses, catheterisation, fibrinolytics, and anti-inflammatory medications. Rapid diagnosis and surgical repair have been shown to reduce long-term complications such as erectile dysfunction, painful sexual intercourse, deviation of the penis, and urethral injury from 40% to 11% compared to conservative management alone [16].

## 4. Conclusion

Penile fracture is a rare urological emergency diagnosis, and presentation is often delayed. Recurrent fracture has only been documented in a handful of cases, with only two prior documented recurrent contralateral penile fractures on literature review. Rapid diagnosis and surgical intervention must be the priority in case management to minimise long-term morbidity, with particular attention to be paid to ensuring equal corporal lengths intra- and postoperatively to prevent recurrence.

## References

[1] Morey A. F., Rozanski T. A. (2007). Genital and lower urinary tract trauma. *Campbell-Walsh Urology 9th edition Volume 3, Chapter 83*.

[2] Reis L. O., Cartapatti M., Marmiroli R. (2014). Mechanisms predisposing penile fracture and long-term outcomes on erectile and voiding functions. *Advances in Urology*.

[3] Koifman L., Cavalcanti A. G., Manes C. H., Filho D. R., Favorito L. A. (2003). Penile fracture: experience in 56 cases. *International Brazilian Journal of Urology*.

[4] Kattan S., Yaussef A., Onuora V., Patil M. (1993). Recurrent ipsilateral fracture of the penis. *Injury*.

[5] Shabir A. M., Mumtaz D. W. (2013). Recurrent penile fracture. *World Journal of Medical and Surgical Case reports*.

[6] El Malik E. F., Ghali A. M., Ibrahim A. I., Rashid M. (1997). Fracture of the penis: a critique of clinical features and management. *Annals of Saudi Medicine*.

[7] Nicolaisen G. S., Melamud A., Williams R. D., McAninch J. W. (1983). Rupture of the corpus cavernosum: surgical management. *The Journal of Urology*.

[8] Muentener M., Suter S., Hauri D., Sulser T. (2004). Long-term experience with surgical and conservative treatment of penile fracture. *The Journal of Urology*.

[9] Kitrey N. D., Djakovic N., Hallscheidt P. (2021). *“EAU guidelines on urological trauma”. Edn. presented at the EAU Annual Congress Milan*.

[10] Mazaris E., Livadas K., Chalikopoulos D., Bisas A., Deliveliotis C., Skolarikos A. (2009). Penile fractures: immediate surgical approach with a midline ventral incision. *BJU International*.

[11] Kamdar C., Mooppan U., Kim H., Gulmi F. (2008). Penile fracture: preoperative evaluation and surgical technique for optimal patient outcome. *BJU International*.

[12] Brandes S. (2006). Initial management of anterior and posterior urethral injuries. *Urologic Clinics of North America*.

[13] Sharma S., Suryavanashi M., Sharma S., Singh S., Sethi A., Gupta N. P. (2009). Contralateral fracture of the penis with concomitant urethral injury — report of a rare case. *African Journal of Urology*.

[14] Dell'Atti L., Scarcella S., Tallè M., Polito M., Galosi A. (2019). Simultaneous curvature correction at the time of the penile fracture repair: surgical and functional outcomes. *Research and Reports in Urology*.

[15] Mbonu O. O., Aghaji A. E. (1992). Fracture of the penis in Enugu, Nigeria. *Journal of the Royal College of Surgeons in Edinburgh*.

[16] Gamal W. M., Osman M. M., Hammady A., Aldahshoury M. Z., Hussein M. M., Saleem M. (2011). Penile fracture: long-term results of surgical and conservative management. *Journal of Trauma*.

